# Compound Probiotics Improve the Diarrhea Rate and Intestinal Microbiota of Newborn Calves

**DOI:** 10.3390/ani12030322

**Published:** 2022-01-28

**Authors:** Bo Liu, Chunjie Wang, Simujide Huasai, Aricha Han, Jian Zhang, Lina He, Chen Aorigele

**Affiliations:** 1 College of Animal Science, Inner Mongolia Agricultural University, Hohhot 010018, China; lb15754882650@163.com (B.L.); smjd_2010@163.com (S.H.); 15248146527@163.com (A.H.); ZhangJian280613@163.com (J.Z.); na15374712742@163.com (L.H.); 2 College of Veterinary Medicine, Inner Mongolia Agricultural University, Hohhot 010018, China; chunjiewang200@sohu.com

**Keywords:** newborn calves, compound probiotics, gut microbiome, 16S rRNA sequencing, diarrhea

## Abstract

**Simple Summary:**

Calf diarrhea is a major cause of mortality in calves, and results in high treatment costs and economic loss for the dairy and cattle industries. In addition, diarrhea usually occurs around 2 weeks after calf birth. In this study, we determined how compound probiotics influenced the gut microbiota and its effect on diarrhea rates of newborn Holstein calves. The probiotics included compound yeast (*Saccharomyces cerevisiae* and *Kluyveromyces marxianus*) and lactic acid bacteria (*Lactococcus lactis* subsp. *lactis*, *Pediococcus pentosaceus*, and *Lactobacillus plantarum*). Among them, the LS, L, and S groups are different compound probiotic groups, and the D group is the control group. Our results revealed that although probiotics did not affect the community diversity of gut bacteria in newborn calves, compound probiotics significantly increased the community richness of gut bacteria. Principal coordinates analysis using weighted UniFrac distances showed that the microbial communities of calves fed compound probiotics were relatively closely clustered, but were separate from the communities of calves in the control group. The calves fed compound probiotics also had lower rates of diarrhea. Our findings improve our understanding of the role of probiotics in regulating the gut microbiota of calves, and are of special significance to researchers in the dairy and cattle industries.

**Abstract:**

We evaluated the effects of probiotic compounds on the composition of the gut microbiota. Forty newborn calves were random allocated to the lactic acid bacteria + yeast group (LS group), lactic acid bacteria group (L group), yeast group (S group), and control group (D group). Probiotics containing *Lactococcus lactis* subsp. *lactis*, *Pediococcus pentosaceus*, *Lactobacillus plantarum*, *Saccharomyces cerevisiae*, and *Kluyveromyces marxianus* were fed to calves in the three treatment groups for 15 days. The feeding process lasted 15 days. Fecal samples were collected from all calves at the end of the trial and analyzed using high-throughput 16S rRNA sequencing. Totals of 1,029,260 high-quality reads and 420,010,128 bp of sequences were obtained. Among the four groups, the alpha diversity of gut microbes was significantly higher in newborn cattle in the LS group than in those in the L, S, and D groups. Overall, the dominant phyla were *Firmicutes*, *Actinobacteria*, and *Bacteroidetes*, whereas *Bifidobacterium* was the most abundant phylum in the gut of cattle in the LS group. Newborn calves from the compound probiotic groups had closely clustered gut bacterial communities and had lower rates of diarrhea. Overall, compound probiotics regulated the intestinal microbiota community structure of newborn calves and improved intestinal health. New information relevant to the prevention of diarrhea is provided by our research in newborn calves.

## 1. Introduction

For humans and animals, diarrhea is a common disease, which is frequently associated with disorders of intestinal flora and damage to the intestinal mucosal barrier. Bacterial invasion, parasitic infection, dietary changes, and viral infection can all cause diarrhea. Thus, diarrhea is common in calves, and causes high morbidity and mortality. In later stages, diarrhea also seriously affects the growth, development, and health of calves, which leads to high treatment and breeding costs to the breeding industry and results in considerable economic loss. Therefore, the effective prevention and treatment of diarrhea in calves is conducive to the development of the breeding industry. Intestinal microorganisms play a significant part in animal intestinal health and perform various functions, including degradation of carbohydrates and fiber, regulation of dietary lipid intake and deposition, production of certain vitamins and short-chain fatty acids, appropriate stimulation of the immune system, regulation of intestinal movement, and protection of hosts from intestinal pathogens [1].

Imbalances in intestinal absorption, movement, and secretion caused by imperfect intestinal function in newborn calves are associated with disturbances in intestinal flora [2]. After birth, newborn calves obtain numerous microorganisms from the external environment, which colonize the gastrointestinal tract. Once the intestinal microbial barrier is breached, a large number of pathogenic bacteria colonize the intestinal tract, thus causing inflammation [3]. Multiple enteric pathogens, such as viruses and bacteria, are involved in the development of diarrhea [4]. Therefore, we examined the potential influence of probiotics on the gut microbiome of newborn calves.

The health and growth of dairy cows are known to be associated with the fecal microorganisms and early intestinal microbial composition of pre-weaning calves [5]. Lee et al. found that combined feeding of *Lactobacillus plantarum* and *Bacillus subtilis* can reduce the duration of calf diarrhea, help balance the intestinal flora, and can even prevent calf diarrhea [6]. Furthermore, the abundance of *Clostridium perfringens* increases significantly in the intestines of diarrheic dogs, indicating that this enterotoxin-producing pathogen may cause animal diarrhea related to disorders of the intestinal flora [7]. Kawakami et al. reported that feeding milk replacers containing yeast and lactic acid bacteria to calves significantly increased their feed conversion rate, reduced the fecal score, and inhibited diarrhea [8]. An et al. also found that lactic acid bacteria can regulate the balance of gastrointestinal flora in calves, reduce the number of pathogenic bacteria in their feces, improve their health, and reduce diarrhea [9]. The same study also reported that the probiotics colonized the intestinal tract of the animals and adhered to the surface of the intestinal mucosa, thus forming a microbial protective layer to prevent the invasion of pathogenic bacteria and reduce their living space. Intestinal bacteria also produce short-chain fatty acids—such as propionic acid, lactic acid, and acetic acid—that enhance intestinal acidity, thus limiting the living environment of pathogenic bacteria [10]. These findings suggest that probiotics are excellent candidates for preventing diarrhea. Therefore, it is important to study the alterations in gut microbiome composition to easily comprehend the probiotic–host interactions in disease and health.

The purpose of this study was to describe the beneficial effects of compound probiotics in improving the gut microbiota structure and reducing the rate of diarrhea in newborn calves. We used 16s rRNA sequencing to investigate changes in gut microbial diversity and structure in newborn calves following the addition of complex probiotics. Our results suggest that the addition of complex probiotics can improve the gut microbiota structure of newborn calves and reduce the rate of diarrhea at this stage.

## 2. Materials and Methods

### 2.1. Animals and Sample Collection

All calves were born in the Qishi pasture, Ordos city, Inner Mongolia Autonomous Region, China. Each calf was fed 4 L of colostrum immediately 1 h after birth, and 6 h later, each calf was fed 2 L of colostrum. After another 6 h, the calves were fed 2 L of colostrum. The colostrum came from each calf’s own dam. Then, all calves were transported to calf islands (1.2 m × 3 m) and were fed with 3 L of milk substitute (Eastern Bell, Beijing, China) at a constant 38 °C to 40 °C at 06:00 and 17:00 daily. The concentration of milk substitute was 125 g/L. During this period, the calves were free to drink water and eat concentrates (Borui, Inner Mongolia, China). We randomly selected 40 healthy newborn Holstein calves and divided them into four groups according to their age and weight (age: 3.21 ± 0.56 days old; weight: 36.3 ± 2.03 kg), with 10 calves in each group. The groups were as follows: lactic acid bacteria + yeast group (group LS), lactic acid bacteria group (group L), yeast group (group S), and placebo group (group D). Calves in the LS, L, and S groups were fed daily with milk substitute supplemented with 100 mL (2 × 10^8^ CFU/mL) of compound lactic acid bacteria + yeast, compound lactic acid bacteria, and compound yeast, respectively. Group D was fed only 100 mL of a placebo containing the growth medium without probiotics. The yeast probiotics included Saccharomyces cerevisiae and Kluyveromyces marxianus (1 × 10^8^ CFU/mL each). The lactic acid bacteria included Lactococcus lactis subsp. lactis, Pediococcus pentosaceus, and Lactobacillus plantarum (1.3 × 10^10^ CFU/mL each). The lactic acid bacteria + yeast probiotics contained the five kinds of bacteria mentioned above. All five probiotics were selected from koumiss (a fermented dairy product) in our laboratory. During the trial period, calves receiving the rehydration treatment for diarrhea were not fed any antibiotics. Rehydration was based on fecal score. For calves who needed rehydration therapy, an intravenous injection of glucose and sodium chloride injection (glucose concentration of 5%, sodium chloride concentration of 0.9%) and a vitamin C injection (vitamin C concentration of 90%) were provided. The glucose and sodium chloride injection was provided by the Shannxi Shengao Animal Pharmaceutical Co., Ltd. (Xianyang, China). The vitamin C injection was provided by the Chengdu Xinheng Pharmaceutical Co., Ltd. (Chengdu, China). Rehydration treatment was administered by the ranch veterinarian. During the trial, the calves had no other infections. In addition, we did not test for the main infectious causes of diarrhea (rotavirus and corona virus, *E. coli*, Cryptosporidium, etc.), because we could not estimate how specific pathogens would interact with the microbial community. The nutrient contents of the milk substitute and calf starter are listed in Table 1. At the end of the trial, fecal samples were gathered from each calf and stored in sterile tubes immediately after normal defecation. The samples were then sent to the laboratory in liquid nitrogen and stored at −80 °C until DNA extraction.

### 2.2. Weight, Diarrhea Rate, and Fecal Score

In the early morning of the first day of the experiment, when the calves did not have any food, their body weight was measured as the initial body weight. The final weight was measured at the end of the trial, and the average daily gain (ADG) of each calf was calculated.

The fecal condition of each calf was observed and scored every morning. This process was done by a dedicated person who knew what the different groups of calves were. If the fecal matter had a normal shape, but was not hard, and the shape changed slightly after falling to the ground, it was marked 1 point. If the fecal matter was soft, piled up, and the shape was difficult to maintain, it was marked as 2 points. If the fecal matter had the consistency of pancakes or ointment and was easy to spread, it was marked as 3 points. Finally, if it had the consistency of water and showed solid–liquid separation, it was marked as 4 points. Cases with ≥3 points were recorded as diarrhea, and the rate of diarrhea was calculated after the measurements had been completed. The formula for calculating the diarrhea rate is as follows:

Diarrhea rate (%) = sum of diarrhea calves per day/total number of calves × examined days × 100 (1)

### 2.3. DNA Extraction and Sequencing

The microbial community’s genomic DNA was separated from fecal samples employing the soil DNA kit (Omega Bio-tek, Norcross, GA, USA) following the manufacturer’s instructions. The DNA was electrophoresed on a 1% agarose gel and its concentration and purity were calculated. These protocols have been previously described [11]. The bacterial 16S rRNA gene’s hypervariable V3-V4 areas were amplified with the primer sequence 338F (5′-ACTCCTACGGGAGGCAGCAG-3′) and 806R (5′-GGACTACHVGGGTWTCTAAT-3′) using an ABI GeneAmp^®^ 9700 PCR thermocycler (Applied Biosystems, Foster City, CA, USA). The 16S rRNA gene’s PCR amplification was carried out according to the design of the Ning et al. [12]. The PCR mixtures contained 4 μL of 5× TransStart FastPfu buffer, 2 μL of 2.5 mM dNTPs, 0.8 μL each of the forward and inverse primers (5 μM each), 0.4 μL of TransStart FastPfu DNA polymerase, 10 ng of template DNA, and ddH2O up to 20 μL. PCRs were carried out in triplicate. A DNA gel extraction kit (Axygen Biosciences, Union City, CA, USA) was used to extract PCR products [12]. Sequencing was performed according to a standard protocol [13].

Relevant software (Illumina, San Diego, CA, USA) was used to process the raw sequence. For detailed steps, please refer to Chen’s method [14]. See references for clustering and classification notes of operational taxonomic units (OTUs) [15].

### 2.4. Statistics Analysis

Statistical analyses were carried out using the PROC MIXED of SAS version 9.4 (SAS Institute Inc., Cary, NC, USA). The statistical model was:

Yij = µ + Di + Cj + εij(2)

where Yij is the observed variable, µ is the overall mean, Di is the fixed effect of different treatment, Cj is the random effect of calves, and εij denotes the residual error. Differences between the means were tested using Bonferroni multiple comparison test. One way ANOVA and LSD multiple comparisons were used to analyze whether the species diversity was significant between groups. Differences were considered to be significant (*p* < 0.05).

## 3. Results

### 3.1. Average Daily Gain, Fecal Score, and Diarrhea Rate

The body weight, ADG, fecal score, and diarrhea rates of calves in different groups are listed in Table 2. Compared with the group LS, L, and S, the final body weight of group D was significantly lower than that of the other three groups (*p* < 0.05). Although the differences in ADG among the groups were not significant, group D was also lower than the other groups. There were no significant differences in fecal scores between groups (*p* > 0.05). However, the fecal score group D was higher than the other three groups for most of the time of the experiment. The diarrhea rate of calves in group D reached 39%, while the diarrhea rates in groups LS, L, and S were 22%, 28%, and 26% respectively.

### 3.2. 16S rRNA Gene Sequencing and Microbial Diversity

A sum of 1,374,535 raw reads were produced from the 20 fecal samples making use of Il-lumina HiSeq sequencing. When low-quality reads were filtered, 1,029,260 clean reads were selected from 560,904,851 bp of sequences. The average length of these sequences was 408.07 bp. All reads were classified into 383 operational taxonomic units (OTUs) belonging to 72 families and affiliated to 12 phyla (Supplementary Materials Table S1). Good’s Coverage estimates of all samples were 99.99%, indicating that the sequencing depth was adequate to observe the microbial community in the samples [16]. To comfortably comprehend OTU diversity in each group, we compared the alpha diversity among newborn cattle from the different compound probiotic groups. We used the Chao1 and ACE indices and the Simpson and Shannon indices to estimate bacterial community richness and diversity, respectively. The results revealed that there were no considerable contrasts in the Simpson diversity indices and Shannon of the microbial community between the LS, S, L, and D groups (*p* = 0.798 and *p* = 0.253, respectively) (Table 3). However, the ACE and Chao1 richness indices were significantly higher in the LS group than in the other three groups, and were significantly higher in the L and S groups than in group D (*p* < 0.001). Though there were similarities in microbial communities’ diversity among the groups, there were differences in community richness.

### 3.3. Beta Diversity

**Figure 1:**
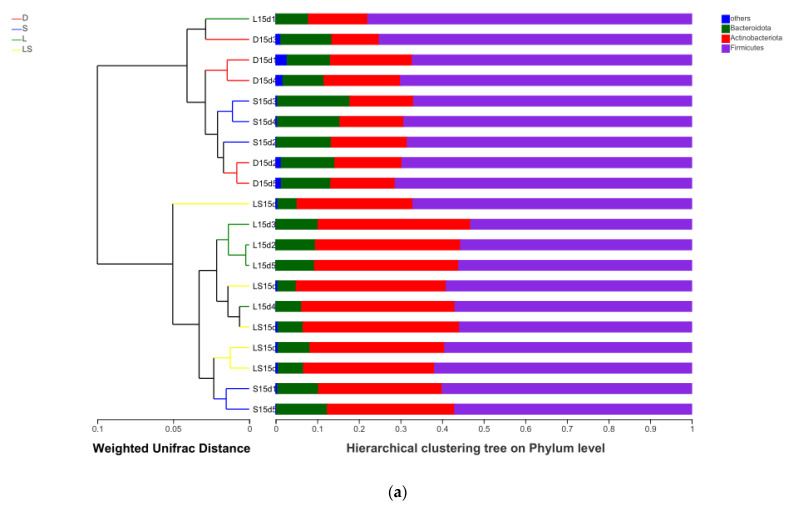

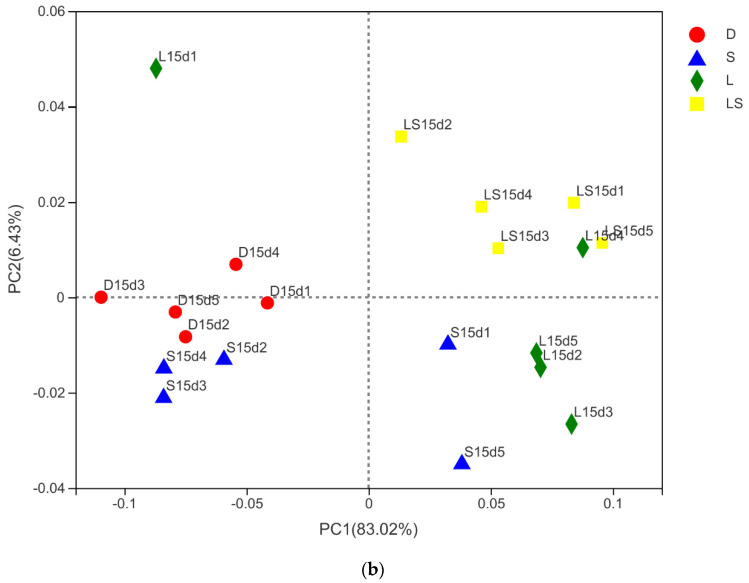
Relationships between the gut microbiota of newborn calves from different probiotic treatment groups. (**a**) Clustering analysis of the evolution of gut microbiotas in newborn calves from the LS, L, S, and D groups (LS group: lactic acid bacteria + yeast group; L group: lactic acid bacteria group; S group: yeast group; D group: control group). Gut microbiota trees were produced making use of the UPGMA algorithm based on the unweighted UniFrac distances generated using QIIME software. (**b**) Principal coordinates analysis of weighted UniFrac distances among the microbial communities of newborn calves in different probiotic groups.

A UPGMA cluster tree based on weighted UniFrac distance was used to further compare the diversity of intestinal microbiota among the different groups. Interestingly, the gut microbiota in the LS, L, and S groups were more clustered, whereas the newborn calves in group D were separated (Figure 1a). Principal coordinates analysis (PCoA) was used to evaluate the gut microbiota’s community composition, and weighted UniFrac distances were worked out based on a phylogenetic tree. When visualized in the PCoA plot, one fecal sample was represented by each symbol (Figure 1b). Similar to the results of the aforementioned hierarchical cluster analysis, the fecal samples of newborn calves in the four treatment groups were clearly separated, although the samples within each group were clustered. The samples in the LS, L, and S groups were clustered in the same area and were separated from the cluster of group D samples along principal coordinate axis 1 (PC1), which explained the highest proportion of variation (83.02%). The results showed that there was little difference between the LS, L, and S groups, and that the gut bacterial communities were similar between these groups.

### 3.4. Bacterial Community Taxonomic Composition

**Figure 2:**
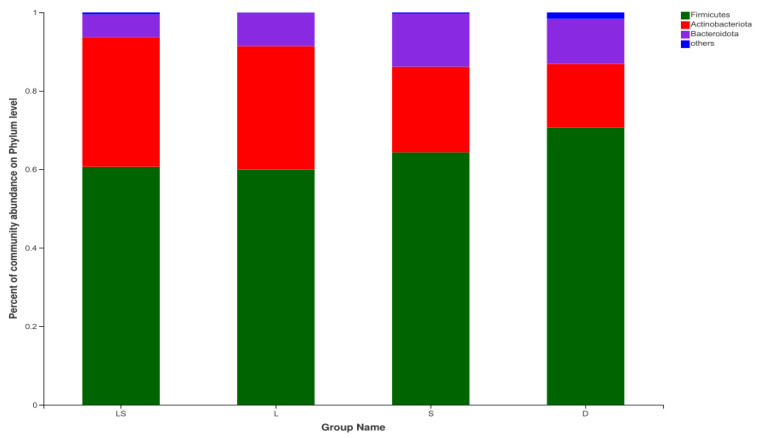
Relative abundance of bacterial groups from the LS, L, S, and D groups at the phylum level. LS group: lactic acid bacteria + yeast group; L group: lactic acid bacteria group; S group: yeast group; D group: control group.

All the sequences obtained from the four groups were classified at the phylum and genus levels. In total, we identified 12 bacterial phyla. Among these, Firmicutes, Actinobacteria, and Bacteroidetes had relatively high abundances, with mean abundance levels of 63.89 ± 4.91% (mean ± standard error of the mean), 25.63 ± 8.01%, and 9.83 ± 3.38%, respectively (Figure 2). The abundance of Actinobacteria in the LS and L groups (33.06% ± 3.83% and 31.46% ± 9.69%, respectively) were higher than those in the S and D groups (21.83 ± 7.70% and 16.19 ± 3.19%, respectively). In contrast, the abundances of Bacteroidetes in the LS and L groups (5.79 ± 1.33% and 8.54 ± 1.55%, respectively) were lower than those in the S and D groups (13.51 ± 2.89% and 11.48 ± 1.29%, respectively). Actinobacteria and Bacteroidetes differed significantly between the four groups (*p* = 0.003 and *p* < 0.001, respectively), whereas Firmicutes was not significantly different between the groups (*p* > 0.05) (Supplementary Materials Table S2).

**Figure 3:**
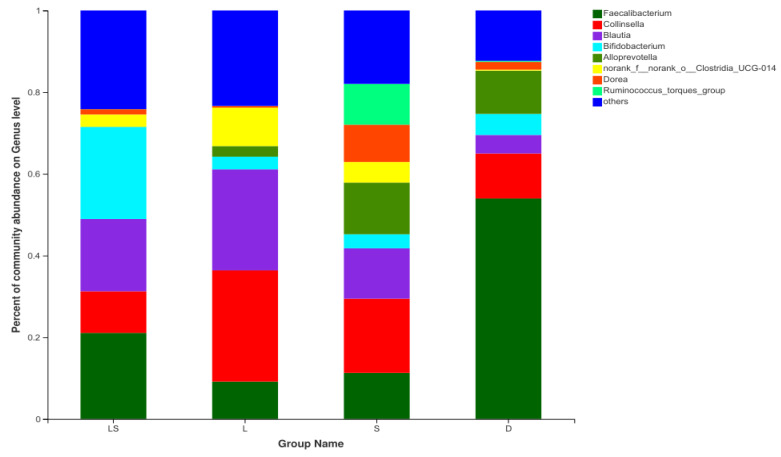
Relative abundance of bacterial groups from the LS, L, S, and D groups at the genus level. LS group: lactic acid bacteria + yeast group; L group: lactic acid bacteria group; S group: yeast group; D group: control group.

At the genus level, 158 genera were detected in all the samples. *Faecalibacterium*, *Collinsella*, *Blautia*, and *Bifidobacterium* were the predominant genera in all the groups. In the LS, L, and S groups, the proportions of *Faecalibacterium* (21.08 ± 3.05%, 9.16 ± 4.06%, and 11.29 ± 4.31%, respectively) were lower than that in the D group (53.98 ± 4.89%, *p* < 0.0001). The proportion of *Bifidobacterium* in the LS group (22.55% ± 2.42%) was higher (*p* < 0.0001) than those in the L, S, and D groups (3.09 ± 0.43%, 3.42 ±0.54%, and 5.16 ± 0.87%, respectively). In the LS, L, S, and D groups, the relative abundances of *Collinsella* were 10.18 ± 4.13%, 27.23 ± 9.15%, 18.15 ± 7.59%, and 11.01 ± 3.19%, respectively; and those of *Blautia* were 17.69 ± 1.14%, 24.73 ± 3.22%, 12.34 ± 3.49%, and 4.52 ± 1.03%, respectively (Figure 3) (Supplementary Materials Table S3).

### 3.5. Microbial Signatures

**Figure 4:**
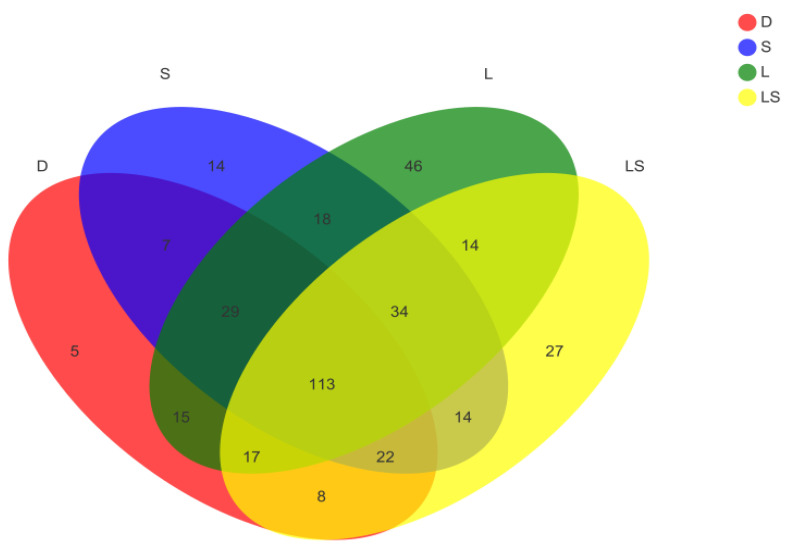
A Venn diagram based on the operational taxonomic units of newborn calves in the different probiotic treatment groups.

**Figure 5:**
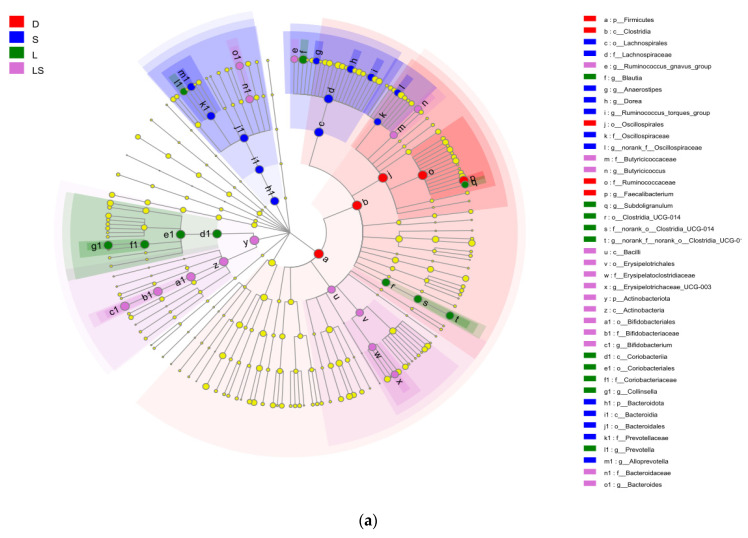

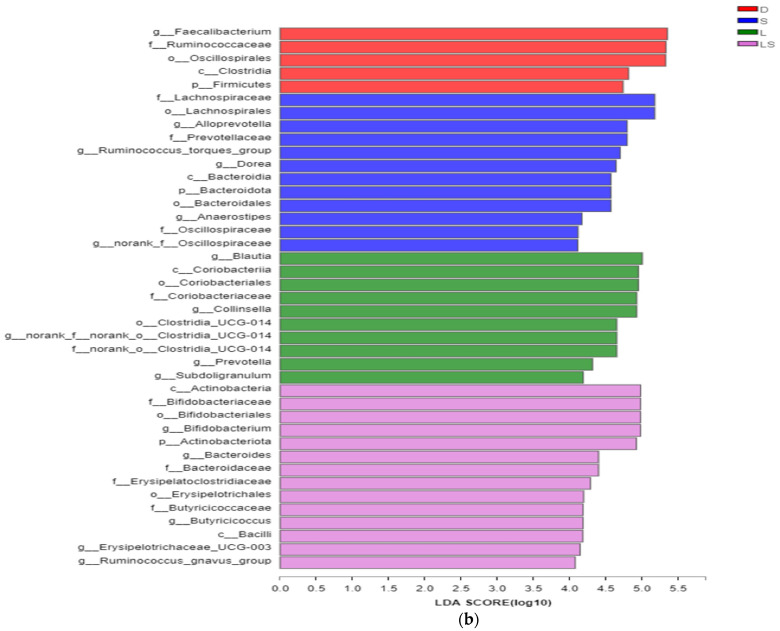
The OTU tables were used on by a linear discriminant analysis effect size algorithm’s consequences to decide the taxa best characterizing each biological class. (**a**) A cladogram that shows bacterial lineages’ phylogenetic distribution in newborn calves from the dissimilar probiotic groups. Different constituents are represented by different colored regions (purple, LS; blue, S; green, L; red, D). Phylogenetic levels are indicated by the circles from the genus level (outer circles) to the phylum level (inner circle). (**b**) LDA scores of the operational taxonomic units in calves from the different probiotic groups. LS group: lactic acid bacteria + yeast group; L group: lactic acid bacteria group; S group: yeast group; D group: control group.

In total, we found 383 OTUs in all 20 fecal samples (Figure 4), of which 27 OTUs were unique in the LS group, 46 in the L group, 14 in the S group, and 5 in the D group. The total number of OTUs common among all four groups was 113. Linear discriminant analysis effect size (LEfSe) analysis was used to decide the taxa that explained the contrasts between newborn calves most likely in the dissimilar probiotic treatment groups. The LEfSe analysis’ consequences confirmed that the phylum *Actinobacteria*, class Actinobacteria, order Bifidobacteriales, family Bifidobacteriaceae, and *Bifidobacterium* were significantly enriched in the LS group; the genera *Blautia* and *Collinsella* were enriched in the L group; the families Lachnospiraceae and Prevotellaceae and the genus *Alloprevotella* were enriched in the S group; and the family Ruminococcaceae and genus *Faecalibacterium* were enhanced in the D group (Figure 5a,b).

## 4. Discussion

The calf stage is known to be a critical period for the growth and development of the gastrointestinal tract in cattle. With the effective colonization of microorganisms in the external environment, the gastrointestinal tract forms a sound internal microbial environment, which is beneficial for the decomposition of the three major dietary nutrients [17]. After birth, calves are weaned after approximately 60 days. The gastrointestinal tract being colonized with a large number of pathogenic bacteria during this time is not conducive to its normal development. Early regulation of the gastrointestinal flora can—to a certain extent—reduce the risks of diarrhea and death in calves [18]. However, studies have shown that calves have the highest chance of developing diarrhea between 1 and 15 days of age [4]. In the present study, feeding the compound probiotics did not result in a significant rise in ADG of the calves in the experimental groups. One possible explanation of this consequence is that milk replacer for calves is either low in daily amount or concentration. However, the ADG of the LS, L, and S groups (0.6, 0.6, and 0.61, respectively) were higher than those of the D group (0.47). The fecal scores were not significantly different between the four groups, but group D (Day 3–6 and Day 9–11) had higher scores than the other three groups during the trial. Group D (39%) had the highest rate of diarrhea of all treatment groups (LS: 22%, L: 28%, and S: 26%). This may be because of the protective effect of probiotics that helps prevent infections by pathogenic bacteria. Intestinal bacteria also produce short-chain fatty acids that enhance the acidity of the intestinal tract and restrict the living environment of pathogenic bacteria [8]. In addition, probiotics have been proven to prevent diarrhea [19].

Probiotics can help balance the community structure of intestinal flora in animals, produce biologically active substances in the intestines, raise the abundance of beneficial bacteria, and reduce the abundance of pathogenic bacteria. Elucidating the effects of probiotics on intestinal microbial diversity can enhance our appreciation of the relationship between probiotics, microbial community structure, and health. In the current study, we found that the gut microbes in cattle from the LS group had higher community richness than that in the other groups. Although the bacterial abundance was lower in the L and S groups than in the LS group, it was higher in all three groups than that in group D. There was no significant difference in microbial diversity between all the groups. This may be because after the calf is born, the digestive functions of its gastrointestinal tract are not fully developed, the types and number of microorganisms in the intestine are very few, and the community structure of the flora is relatively homogenous. The species richness and microbial diversity of the key microbiota is reduced by intensive farming [20]. In addition, since we only selected fecal samples from 20 calves for sequencing analysis, the relatively smaller sample size may also be the reason for the insignificant diversity of flora. At this time, the intestinal flora of the calf is in a stage of development and has a certain degree of plasticity. In this study, the combined action of lactic acid bacteria and yeast significantly increased the abundance of beneficial bacteria in the calf intestine. This indicates that the compound probiotics in the LS group had a better effect on the intestinal flora of calves than lactic acid in the L group or yeast in the S group. This is supported by the report that probiotic interventions promoted the gut microbiota’s maturation through increasing microbial diversity and species richness, which could enhance the digestive and immune systems’ development and build up disease resistance [21]. These results may be further associated with economic benefits for the cattle industry.

The fecal microbiota in newborn cattle in the LS, L, and S groups were clustered relatively closely according to the UPGMA cluster analysis, whereas those in the D group were separated. This may be because probiotics change the community structure of the intestinal flora in newborn calves [22]. The PCoA that used weighted UniFrac distances disclosed that newborn cattle’s intestinal microflora was associated with the probiotics and that samples in the group fed compound probiotics overlapped substantially; however, the control group was clustered independently. In this study, although the composition of the compound probiotics was different between groups, they had comparable effects on the clustering of the intestinal flora of newborn calves. This indicated that probiotics regulated the abundance of probiotics in the intestinal tract of calves with diarrhea, which is beneficial for reducing the risk of calf diarrhea [23].

In this study, the principal bacterial phyla in the gut microbiome were Firmicutes, Actinobacteria, and Bacteroidetes. Former investigations have shown that Firmicutes and Bacteroidetes are some of the main microbial groups in the intestinal flora of healthy calves [24,25]. Furthermore, gut microbial communities with a high abundance of Firmicutes allow cattle to optimize energy absorption from foods to maintain their body functions [26]. Most taxa in Firmicutes are dependent on dietary fiber [27]. The newborn calves in this study did not have fully developed rumen function, did not consume plant-based feed, and were fed a milk replacer as the main energy source; therefore, the abundance of Firmicutes was not significantly different between the probiotic groups and the D group. Actinobacteria—one of the main types of bacteria found in the intestinal tract—plays a central role in maintaining intestinal homeostasis [28]. It is involved in the decomposition of plant-derived carbohydrates and in the body’s inflammation and autoimmune responses [29,30]. In this research, the abundance of Actinobacteria was significantly higher in the L groups and LS than in the D groups and S. The addition of compound lactic acid bacteria + yeast and lactic acid bacteria alone significantly increased the abundance of Actinobacteria in the intestinal tract of newborn calves. We speculate that the high levels of Actinobacteria in the intestine are beneficial for the intestinal upkeep of homeostasis and the absorption of nutrients. At the genus level, the top four relatively abundant genera in the intestinal microbiota were *Faecalibacterium*, *Collinsella*, *Blautia*, and *Bifidobacterium*. *Bifidobacterium* is a beneficial strain that adheres to the surface of the intestinal mucosa, effectively improving the structure of the intestinal flora. Piglets with diarrhea have a significantly lower number of *Bifidobacterium* in their feces [31], and the intake of probiotics has been shown to stimulate the proliferation of *Bifidobacterium* [32]. Due to the invasion and destruction of the intestinal mucosa by pathogenic bacteria, diarrheic calves are unable to effectively absorb nutrients through the intestine. These nutrients are excreted in the form of diarrhea and promote the growth of *Faecalibacterium* in large numbers [33], which may explain the large amount of *Faecalibacterium* in the feces of calves in group D. Finally, *Blautia* is an anti-inflammatory bacterium that produces a bacteriostatic protein which inhibits colonization by pathogenic bacteria [34].

The bacterial communities in the samples from the compound probiotic groups (the LS, L, and S groups) were substantially dissimilar from those in the D group. Although these three groups had different bacterial species compositions, the probiotics had comparable effects in regulating the community structure of the intestinal flora of newborn calves. Overall, the abundance of beneficial bacteria was increased by the probiotics. Calves in the lactic acid bacteria + yeast group possessed a significantly higher abundance of beneficial bacteria in their intestinal tract, in comparison with the calves in the compound lactic acid bacteria and compound yeast groups. These results strongly suggest that probiotic factors affect the composition of gut microbiota in cattle.

## 5. Conclusions

In this study, the intestinal flora in the cattle from the compound probiotic groups were clustered more closely than those from the group without probiotics, and calves in the compound probiotic group had lower rates of diarrhea. Moreover, feeding with compound lactic acid bacteria + yeast increased the abundance of beneficial bacteria in the intestinal tract of newborn calves. Thus, our findings improve our understanding of the effect of compound probiotics on newborn calves’ intestinal flora. However, the manner in which the compound probiotics play a role in preventing diarrhea in newborn calves needs to be further studied through follow-up experiments.

## Figures and Tables

**Table 1:** Nutrient levels of the milk substitute and calf starter after air-drying.

Items ^1^	Content of Milk Substitute ^2^	Content of Calf Starter
Dry matter (%)	90.16	87.2
Crude protein (%)	22.6	20.6
Ether extract (%)	17.8	4.56
Ash (%)	5.62	8.06
Crude fiber (%)	0.18	10.89
Calcium (%)	0.71	1.12
Phosphorus (%)	0.61	0.51

^1^ Calculated. ^2^ Milk substitute lactose levels, 38–46%; V A ≥ 5000 IU/kg; NaCl ≥ 1%; Fe ≥ 50ppm; Cu ≥ 5ppm. The ingredients of the milk substitute: whey protein powder, coconut oil, palm oil, VA, etc.

**Table 2:** Effect of compound probiotics on body weight, average daily gain (ADG), fecal score, and diarrhea rate in newborn calves.

Item	Groups ^1^				SEM	*p*-Value
	LS	L	S	D		
Initial body weight (Kg)	36	36.3	36.4	36.6	1.93	0.053
Final body weight (Kg)	45.23 ^a^	45.03 ^a^	45.46 ^a^	43.69 ^b^	2.19	0.049
ADG (Kg/day)	0.6	0.6	0.61	0.47	0.13	0.227
Fecal score						
Fecal score day 1	2.4	2.3	2.3	2.2	0.46	0.202
Fecal score day 2	2.4	2.2	2.3	2.3	0.46	0.202
Fecal score day 3	2.4	2.5	2.5	2.6	0.68	0.272
Fecal score day 4	2.4	2.4	2.6	3	0.74	0.286
Fecal score day 5	2.3	2.1	2.3	3.1	0.68	0.277
Fecal score day 6	2.2	2.2	2.1	2.4	0.42	0.19
Fecal score day 7	2.1	2.2	2.1	2.1	0.33	0.158
Fecal score day 8	2.2	2.1	2.2	2.1	0.36	0.168
Fecal score day 9	2.2	2.2	2.1	2.4	0.42	0.19
Fecal score day 10	2.5	2.3	2.6	2.8	0.63	0.25
Fecal score day 11	2.3	2.3	2.3	2.9	0.59	0.244
Fecal score day 12	2.3	2.2	2.3	2.2	0.44	0.195
Fecal score day 13	2.3	2.2	2.2	2.2	0.42	0.19
Fecal score day 14	2.2	2.2	2.2	2.3	0.42	0.19
Fecal score day 15	2.2	2.3	2.2	2.7	0.58	0.247
Diarrhea rate (%)	22	28	26	39		

^a,b^ Means with different superscript letters in the same row differ significantly (*p* < 0.05); means with the same or no superscript letters in the same row are not significantly different (*p* > 0.05). ^1^ LS group: lactic acid bacteria + yeast group; L group: lactic acid bacteria group; S group: yeast group; D group: control group.

**Table 3:** Alpha diversity indices of newborn cattle in groups fed different compound probiotics. Data in the table are the mean ± standard error.

Group ^1^	Community Diversity		Community Richness		Goods_Coverage (%)
	Shannon	Simpson	Chao1	ACE	
LS	0.86 ± 0.042 ^a^	0.48 ± 0.028 ^a^	9.67 ± 1.24 ^a^	10.21 ± 1.39 ^a^	99.99 ± 0.001 ^a^
L	0.86 ± 0.108 ^a^	0.48 ± 0.086 ^a^	7.20 ± 2.16 ^b^	7.26 ± 1.43 ^b^	99.99 ± 0.001 ^a^
S	0.89 ± 0.046 ^a^	0.49 ± 0.039 ^a^	7.00 ± 1.22 ^b^	7.64 ± 1.13 ^b^	99.99 ± 0.001 ^a^
D	0.85 ± 0.054 ^a^	0.54 ± 0.033 ^a^	5.00 ± 0.71 ^c^	5.60 ± 0.89 ^c^	99.99 ± 0.001 ^a^
*p*-Value	0.798	0.253	<0.001	<0.001	0.99

^1^ LS group: lactic acid bacteria + yeast group; L group: lactic acid bacteria group; S group: yeast group; D group: control group. ^a,b,c^ Different shoulder letters in the same column indicate significant difference (*p* < 0.05), while the same letters in the same column indicate insignificant difference (*p* > 0.05).

## Data Availability

The datasets that is applied and/or analyzed throughout the prevailing research are available from the corresponding writer upon fair appeal.

## Supplementary Materials

The following supporting information can be downloaded at: https://www.mdpi.com/article/10.3390/ani12030322/s1, Table S1: OTU table of the 20 samples, including the OTU species and sequence number contained in each sample, as well as species annotation information for each OTU. Table S2: The relative abundance of 20 samples at the phylum level. Table S3: The relative abundance of 20 samples at the family level.
